# Immunogenicity and Clinical Efficacy of Influenza Vaccination in Pregnancy

**DOI:** 10.3389/fimmu.2015.00289

**Published:** 2015-06-04

**Authors:** Alexander W. Kay, Catherine A. Blish

**Affiliations:** ^1^Blish Laboratory, Department of Pediatrics, Stanford University School of Medicine, Stanford, CA, USA; ^2^Blish Laboratory, Department of Medicine and Stanford Immunology, Stanford University School of Medicine, Stanford, CA, USA

**Keywords:** influenza, pregnancy, inactivated influenza vaccine, immunogenicity, antibody

## Abstract

Pregnant women are at high risk from influenza due to disproportionate morbidity, mortality, and adverse pregnancy outcomes following infection. As such, they are classified as a high-priority group for vaccination. However, changes in the maternal immune system required to accommodate the allogeneic fetus may alter the immunogenicity of influenza vaccines. A large number of studies have evaluated the safety of the influenza vaccine. Here, we will review available studies on the immunogenicity and efficacy of the influenza vaccine during pregnancy, focusing on both humoral and cellular immunity.

## Introduction

Pregnant women are at increased risk of severe disease secondary to influenza infection, particularly during influenza pandemics. In the 1918 influenza pandemic, maternal mortality was 27% (1), and in one report from the1957 pandemic, half of the fatal cases in reproductive-aged women were among those that were pregnant (2). Rates of hospitalization were higher among pregnant than non-pregnant reproductive-aged women during the pandemics of 1918, 1957, and 2009 (1–3). Though the increased rate of hospitalization in pregnant women is substantially less pronounced during non-pandemic years, pregnant women are still at increased risk from seasonal influenza (4, 5), particularly during the third trimester (6).

As early as 1962, the U.S. Public Health Service identified pregnant women as a priority group for influenza vaccination. However, questions then and now have been raised about how pregnancy alters the quality of the immune response to influenza vaccination. It is generally believed that alterations in immune function contribute to increased influenza severity during pregnancy. Logically, it has also been hypothesized that vaccination during pregnancy may result in a less favorable immunologic response. Immunomodulation during pregnancy has been the subject of several recent reviews (7–10). Here, we will focus, instead, on reviewing the history and evidence on the immunogenicity of the influenza vaccine during pregnancy and its clinical efficacy.

## Assessment of Immunogenicity

To place the existing studies in context, a brief review of the methods used to assess the immune response to vaccination, or immunogenicity, is warranted. The “gold standard” method is the hemagglutinin inhibition (HI) titer, which measures the concentration of antibody required to prevent influenza from agglutinating red blood cells. Thus, the HI titer is a measure of the total amount of antibodies to the hemagglutinin (HA) protein. The WHO defines a “protective” titer as 1:40, based on a 50% reduction in disease, and thus the term seroprotection refers to those individuals with a titer of 1:40 or better (11). Seroconversion is defined as an increase in HI titer following vaccination of fourfold or greater. Virus microneutralization (VMN) assays measure the ability of antibodies in serum to prevent a specific strain of influenza from infecting a cell line, typically Madin–Darby canine kidney epithelial cells. This assay therefore measures the functional capability of antibodies at a specific dilution, rather than just the total quantity. In settings of impaired host immunity, such as HIV infection, the VMN titer is more sensitive than the HI titer (12); VMN is also better for detecting antibodies to avian influenza viruses (13). Multiple studies have demonstrated good correlation between HI titers and VMN titers in pregnant women following monovalent H1N1 vaccination (14, 15), and now seasonal influenza vaccination (16).

## Immunogenicity of Influenza Vaccine in Pregnant Women

In 1962, when a resurgence of the 1957 A2 pandemic influenza strain was anticipated, the U.S. Public Health Service identified pregnant women as a priority group for vaccination based on their historically poor outcomes. However, due to concerns that the same immune alterations that led to increased morbidity could compromise the immune response to the vaccine during pregnancy, Hulka compared the immunogenicity of the vaccine between pregnant and non-pregnant women (17). This placebo-controlled clinical trial compared immune responses after two doses of inactive polyvalent influenza vaccine containing 200 U of A2 antigen and placebo. Overall, in those receiving the vaccine, pooled and individual complement fixation titers were similar between pregnant and non-pregnant women. Further, pooled titers in the non-vaccinated groups were also similar, though they rose at a later time point following circulation of influenza virus in the community. Interestingly, in this study, there was only a marginal, and not-statistically significant, decrease in the rates of influenza-like illness in those receiving the vaccine, whether pregnant or not. While these results indicate that pregnancy did not appear to compromise the immunogenicity of the vaccine, Hulka also did not observe increased disease severity in pregnant women during the 1962 season (17). Consistent with these results, there was no evidence of increased morbidity or mortality in pregnant women from 1958 to 1962 (18). There was also no evidence of increased morbidity and mortality during the 1968 pandemic, which had variable global penetration (19). Thus, the risks posed to pregnant women by influenza may differ according to the circulating strain in a given year. This is an important consideration in evaluating immunogenicity, because it remains possible that differences in the immune response to vaccination could also differ based on vaccine strain.

Without clear evidence for increased morbidity and mortality among pregnant women in 1960s, influenza vaccination of pregnant women during seasonal epidemics was deemphasized as a public health approach until 1976–1977, when a novel influenza strain with pandemic potential emerged at Fort Dix, NJ, USA. The influenza outbreak was ultimately confined to the military base, but in preparation for its spread, approximately 25% of the U.S. population was vaccinated with a novel monovalent A/New Jersey/8/76 (Hsw1N1) vaccine. Vaccine responses to this novel monovalent vaccination were compared between pregnant women and non-pregnant women by HI titers and by using 2-mercaptoethanol treatment to assess the amount of IgM antibody (20). As with the prior study by Hulka, no significant differences between pregnant and non-pregnant women were observed in the geometric mean titers (GMT) following vaccination, nor was there a significant difference in the mercaptoethanol IgM reduction indicative of antigen-specific IgM. Together, these two studies suggested that pregnant women had vaccine responses on par with those of non-pregnant women.

In 1990s and early 2000s, increased attention was given to vaccination of pregnant women. In 1997, the American Committee on Immunization Practice recommended seasonal influenza vaccination for pregnant women in the second and third trimesters, and in 2004, this recommendation was modified to include all pregnant women. In 2008, a large randomized-controlled trial of vaccination in pregnant women in Bangladesh demonstrated that influenza vaccination was clinically efficacious in preventing influenza in pregnant women and their infants (21). The immunogenicity data from this trial were subsequently released in 2010 (22). In this study, there was no non-pregnant control group, but the pregnant women had significant increases in their GMTs to the H1N1, H3N2, and influenza B strains following vaccination, and seroconversion rates of 83.6% for H1N1, 69.2% for H3N2, and 39.7% for B influenza strains (22), which are similar to those seen among healthy adults receiving seasonal influenza vaccination.

Early reports that pregnant women were experiencing disproportionate morbidity and mortality during the 2009 H1N1 pandemic prompted renewed interest in the immunogenicity of the inactivated influenza vaccine (IIV) in pregnant women (3). Multiple studies evaluated immune responses to the monovalent pH1N1 vaccination during pregnancy. Ohfuji et al. enrolled 150 pregnant women receiving the thimerosal-free monovalent pH1N1 (15 μg) vaccination during pregnancy (23). Immune responses were tested by HI titers 3 weeks after the first dose and 4 weeks after the second dose, controlling for body mass index, age, and the receipt of the 2009 seasonal influenza vaccination. Robust responses were noted to the initial vaccination, with an average HI antibody increase of more than 10-fold and a seroconversion rate of 91%. The second vaccination conferred little additional benefit. Importantly, it was noted that receipt of seasonal influenza vaccination <19 days prior to pH1N1 vaccination significantly reduced the fold increase in titer (*p* = 0.021).

A similar study by Tsatsaris et al. enrolled 110 women equally divided between the second and third trimester of pregnancy who received a single dose of monovalent pH1N1 containing 15 μg of HA (15). Subjects were evaluated with HI and VMN assays pre-vaccine and at 21 and 42 days after vaccination. Infant cord-blood titers were also assessed. Pregnant women responded robustly in this study as well: 21 days post-vaccination 98% of women had HI titers of >1:40 (seroprotection) and 93% had seroconverted. It was again noted that women with prior seasonal influenza vaccination had lower fold increases in GMT. In this study, a lower HI GMT was observed in women with twin pregnancy (*p* = 0.006), although it is unclear whether this is secondary to decreased production or increased placental transfer of antibody. Maternal and cord-blood titers were highly correlated (*r* = 0.86). Lastly, VMN titers correlated significantly with HI titers (*r* = 0.96).

Jackson et al. also evaluated responses to the monovalent H1N1 vaccine in pregnant women, investigating whether pregnant women responded differently to a vaccination containing 49 μg HA as compared to 25 μg HA (14). HI and VMN assays were again used to evaluate vaccine responses. Following the first vaccination, 93% of women had titers of >1:40 (seroprotection). The second vaccination did not significantly increase antibody titers, and there was no benefit to using the higher antigen content vaccine. As with Tsatsaris et al., VMN and HI responses were significantly correlated (*r* = 0.81) following vaccination. However, unlike the other two studies, prior receipt of seasonal influenza vaccination was not associated with decreased responses to the monovalent vaccine. Zuccotti et al. published a study of adjuvanted pH1N1 in pregnant women that resulted in titers consistent with seroprotection in 100% of pregnant women, but did not include a pre-vaccine timepoint (24). Overall, though none of these studies included non-pregnant women, the percentage of pregnant women seroconverting and achieving protective titers was similar to studies of the pH1N1 monovalent vaccine performed on non-pregnant men and women (25, 26). However, comparisons with cohorts including men and women are limited by the fact that non-pregnant women respond more robustly than men to influenza vaccination (27, 28). Despite this caveat, as a whole, these studies did not reveal any immunologic deficits, as measured by HI and VMN, to the monovalent pH1N1 vaccination in pregnant women.

Several studies have evaluated vaccine responses to modern seasonal IIV in pregnant women, although only two have included non-pregnant women as a control group. Sperling et al. performed a large multiyear study of 239 pregnant or postpartum women vaccinated with the seasonal influenza vaccine between October 2006 and January 2010, in addition to monovalent H1N1 vaccination in 2009–2010 (29). Overall, the timing of vaccination during pregnancy did not significantly alter HI GMT responses, although there was a trend toward lower responses in the first trimester and 6 weeks postpartum. Seroprotection for H3N2 ranged from 65 to 95% and for H1N1 from 75 to 98%, with higher baseline titers and receipt of vaccination in the prior year associated with lower rates of seroconversion. They found that antibody responses were dominated by IgG1 regardless of trimester.

Along similar lines, Madhi et al. evaluated clinical efficacy and immunogenicity of seasonal trivalent IIV in 2011 and 2012 in South African pregnant women between 20 and 36 weeks gestational age compared to placebo. HIV-uninfected pregnant women responded robustly to IIV with high rates of seroprotection to all vaccine strains (30), though responses were not compared to those of non-pregnant women. In the HIV-infected cohort, the percentage of women with seroprotection was lower; however, HI titers in this group may have underestimated vaccine efficacy, which was 70.6% to confirmed influenza infection. VMN assays were not performed but given prior data on HI titers in HIV infection (12), it is not surprising that HI titers may have underestimated efficacy in this group. Importantly, there was no increase in HIV viral load following maternal vaccination.

Schlaudecker et al. compared HI responses to the 2011–2012 seasonal IIV3 between pregnant women (*n* = 29) and non-pregnant women (*n* = 22) of similar ages (31). They found that while pregnant and control women achieved seroconversion and seroprotection at similar rates, pregnant women had lower post-vaccination GMTs to A/California (H1N1) (*p* = 0.027) and A/Perth (H3N2) (*p* = 0.037). This cohort was unique with respect to prior vaccination history in that 97% of pregnant women and 96% of non-pregnant women had received the influenza vaccination in the previous year. This may suggest that pregnant women are less able to mount serologic responses to previously encountered influenza antigens. However, this would seem to contradict epidemiological data suggesting only subtle differences in disease severity following seasonal influenza infection during pregnancy.

Kay et al. evaluated responses to the 2012–2013 seasonal IIV3 in pregnant women (*n* = 20) and non-pregnant women (*n* = 18) of similar ages. The cohorts were matched by age, but the non-pregnant women were more likely to have received prior influenza vaccinations and had higher baseline HI titers. In contrast to the findings of Schlaudecker et al., pregnant women in this study had equivalent post-vaccination HI titers to those of non-pregnant women for pH1N1 and B/Wisconsin and higher HI fold-change, even after controlling for baseline titers, for the pH1N1 and B/Wisconsin strains (*p* = 0.016 and *p* = 0.014, respectively). This study included VMN titers, which significantly correlated with HI titers across all strains tested. Unlike the HI titers, there were no significant differences in VMN response between pregnant and control women after controlling for baseline VMN titer. Further, pregnant women had higher total IgG levels before immunization (*p* = 0.042) but not following vaccination. Thus, no deficit in the quantity or quality of the antibody response to seasonal IIV was noted in pregnant women in this study.

Kay et al. also assessed the induction of plasmablasts, antibody-secreting, activated B cells, in pregnant and non-pregnant women, relying on data collected in the 2010–2011, 2011–2012, and 2012–2013 seasons. Pregnant women had a significantly greater induction of plasmablasts following vaccination than did non-pregnant controls (*p* = 0.03), though this difference was no longer significant when comparing a small number of pregnant and control women from the 2012 to 2013 influenza season alone. In this study, pregnancy remained predictive of increased plasmablast induction after controlling for baseline average HI titer, suggesting an enhanced induction of antibody-secreting B cells during pregnancy.

Overall, these data suggest that the immunogenicity of IIV, based on the induction of antibodies, is similar in pregnant and non-pregnant women. However, differences in vaccine responses to different influenza strains may complicate our ability to assess subtle changes in responses among pregnant women. Further, responses vary based on prior vaccination or exposure, and pregnant women may mount less robust antibody responses to a secondary challenge. The comprehensive data from pH1N1 monovalent vaccination suggests that pregnant women mount robust antibody responses to a novel influenza vaccine.

## Induction of Cellular Responses

Most studies of influenza vaccine immunogenicity have focused on the induction of antibodies as the correlate of protection. However, several recent studies have evaluated cellular immune responses within peripheral blood mononuclear cells (PBMCs). Forbes et al. compared the induction of cytokines by ELISA, cytometric bead array, and mRNA levels between pregnant and non-pregnant women whose PBMCs were cultured for 48 h with pH1N1 (32). Production of interferon protein and mRNA was reduced in pregnant women who were unvaccinated (*N* = 12) compared with healthy controls, suggesting a deficit in interferon induction during pregnancy. However, interferon production normalized in pregnant women that had undergone pH1N1 vaccination during the prior 12 months, suggesting that vaccination could rescue this defect. Supporting the idea that pregnant women had decreased interferon production, expression of the mRNA encoding protein kinase R, an early interferon stimulating gene, was reduced in pregnant women (32). There were no differences between pregnant and non-pregnant women in the expression of genes encoding the toll-like receptor-3 (TLR3), TLR7, and TLR 9, nor was there a difference in the ability of PBMCs from pregnant or non-pregnant women to support viral replication (32). Subsequently, Vanders et al. found that the percentage of plasmacytoid DCs was reduced in pregnant women and that PDL-1, CD86, and HLA-DR are upregulated on plasmacytoid DCs in pregnancy (33). Blocking antibodies to PD1/PDL1 in pH1N1 PBMC cultures from pregnant women resulted in increased production of IFN-α and IFN-γ, suggesting that deficits in interferon production during pregnancy could be rescued by blocking these inhibitory pathways.

Recently, Kay et al. evaluated NK and T cell responses of pregnant women (*n* = 21) and controls (*n* = 29) to pH1N1 and H3N2 infection of PBMCs *ex vivo* for 7 h (34). Consistent with earlier data, pregnant women had lower IFN-γ production, as measured by intracellular cytokine staining, than did non-pregnant women following stimulation of PBMCs with phorbol 12-myristate 13-acetate and ionomycin. However, in response to influenza infection, the NK and T cells from pregnant women had significantly increased MIP-1β production and enhanced polyfunctional NK and T cell responses compared to non-pregnant women. In this study, vaccination did not significantly affect T or NK cell cytokine and chemokine responses in pregnant women or controls. The assay performed in this study was of shorter duration and used a higher multiplicity of infection than did the assay described by Forbes et al. and Vanders et al. In addition, it is likely that both the pregnant and control women in this study could have been either previously infected by or vaccinated against pH1N1.

In addition to intracellular cytokines, researchers have also evaluated the impact of pregnancy on serum cytokines before and after IIV. Christian et al. compared serum levels of IL-6, TNF-α, IL-8, IL-1β, and migration inhibitory factor (MIF) in 28 pregnant women (average weeks gestational age = 28.4) and 28 non-pregnant healthy women immediately prior to IIV and 1, 2, and 3 days following vaccination (35). Baseline levels of IL-8 and MIF were significantly higher in non-pregnant women. There was no difference in pregnant vs. non-pregnant women in IL-6, TNF-α, or IL-1β responses to vaccination. Pregnant women experienced an increase in MIF levels and no change in IL-8 levels, while non-pregnant women had decreases in both post-vaccination. This group also evaluated HI responses pre- and post-vaccination and found no difference in seroconversion or seroprotection between groups.

Overall, additional study of cellular responses is needed to understand how pregnancy modifies these responses, as a clear picture has not yet emerged. Some data would suggest a deficiency in interferon production, yet other inflammatory pathways may be elevated in response to influenza infection and vaccination during pregnancy. These differences could well be a result of the specific cell types being assessed, kinetic variations in the immune response, or disparities in prior exposure to the influenza strains studied.

## Vertical Antibody Transfer

Vertical transfer of maternal antibodies to the fetus is of equal importance when evaluating influenza vaccine immunogenicity in pregnant women. To this end, Sumaya et al. investigated the immunogenicity of the 1976 monovalent A/New Jersey/8/76 (Hsw1N1) influenza vaccine in 26 maternal serum and cord-blood pairs at the time of delivery. A titer of ≥20 by HI was considered protective against influenza in this study. The GMT of newborn cord bloods was 23.6 and 54% of specimens had protective titers. The GMT of maternal serum was 35.8 and 73% had protective titers. Newborn titers were not significantly affected by the trimester of maternal vaccination (second vs. third). However, the antibodies waned in the infants by 3 months following delivery, when only 12% of infants but 92% of mothers had protective titers. The magnitude of the maternal antibody response correlated strongly with the infant’s antibody titer at 3 months of age (*r* = 0.77, *p* < 0.01). Thus, the authors concluded that passive transfer of antibody did occur, though it appeared to be relatively short-lived. A second study by Englund et al. evaluated placental transfer of maternal antibody to tetanus toxoid and seasonal IIV (36). The 13 women vaccinated with seasonal IIV had robust antibody responses to all three strains as measured by ELISA. The infants had comparable levels of antibody at birth with infant/mother antibody ratios of between 94 and 99% for all three strains (36).

In 2009, Tsatsaris et al. evaluated cord-blood titers in addition to maternal immunogenicity and found that maternal and cord-blood titers correlated (*r* = 0.86). Infant titers of 1:40 or greater were observed in 95% [confidence interval (CI) 89–99%] of the 88 cord-blood samples tested, and cord-blood titers were significantly higher than maternal blood titers. Neither gestational age at vaccination nor delivery significantly affected the neonatal seroprotection rates. Similar results were obtained by Jackson et al. (14). Cord-blood HI GMTs were higher than maternal titers at both vaccine doses and significantly so for the 49 μg dose group (*p* = 0.002). In this study, there was a trend toward lower cord-blood titers with longer intervals between the time of vaccination and delivery. In both of these studies, the cord-blood titers were higher than maternal titers, suggesting active transfer of antibodies across the placenta.

**Table 1:** **Studies evaluating IIV and monovalent pH1N1 vaccine responses during pregnancy**.

Reference	Vaccine, years	Study participants	Immune assays and outcomes	Vaccine response	Summary
**IMMUNOGENICITY OF MODERN SEASONAL INACTIVATED INFLUENZA VACCINE (IIV) IN PREGNANT WOMEN**					
Steinhoff et al. (22), Zaman et al. (21)	Seasonal IIV 2004	340 pregnant Bangladeshi women in the third trimester	HI titers pre- and post-vaccination. Influenza disease endpoints	Seroprotection for H1N1 88%, H3N2 98%, and B 45%. Ratio of maternal to infant titers at delivery ranged from 0.7 to 1.7	High rates of seroconversion and seroprotection following IIV in pregnant women. Reduction in febrile respiratory illness in mothers. Reduction in laboratory-confirmed influenza in infants
Sperling et al. (29)	Seasonal IIV 2006–2010 and H1N1 vaccination 2009–2010	239 pregnant women (73 first, 183 second, 142 third Trimester, 73 immediately postpartum, 36 6 weeks postpartum	HI Titers pre- and post-vaccination to influenza A strains	Seroprotection for H3N2 varied from 65 to 95% and between 75 and 98% for H1N1 strains.	No significant difference in seroprotection or seroconversion by trimester or postpartum. No differences in IgG subtype production in pregnancy vs. postpartum
			IgG subtyping pre- and post-vaccination	
Christian et al. (35)	Seasonal IIV, 2011–2012	28 pregnant women (average gestational age 28.4 weeks) and 28 non-pregnant women	Serum cytokines (prior to, 1, 2, and 3 days post-vaccination) and HI titers pre- and 1 month post-vaccination	Seroprotection rates in pregnant vs. control for H1N1 (89 vs. 85%), H3N2 (81 vs. 93%), and B (83 vs. 100%) were not-statistically different. There were also no significant differences in seroconversion	High rates of seroprotection and seroconversion were observed in both groups. There was not a significant effect observed secondary to vaccination in the prior year. See text for a review of the cytokine responses
Schlaudecker et al. (31)	Seasonal IIV, 2011–2012	29 pregnant women, all trimesters, 22 non-pregnant women	HI titers pre- and post- vaccination	Seroprotection H1N1 93–100%, H3N2 100%, B 58.6–68.2%. Post-vaccination H1N1 GMT 152.53 pregnant vs. 300.46 control, H3N2 GMT 142.0 pregnant vs. 241.0 control	No difference between pregnant and control groups in seroprotection, seroconversion, or fold increase. Significantly increased post-vaccination titers to H1N1 and H3N2 in control women
		Greater than 96% of participants received vaccine in the prior year	
Madhi et al. (30)	Seasonal IIV, 2011–2012, 2012–2013	2116 pregnant women were enrolled, 1062 received IIV, and 1054 received placebo. All trimesters included. An HIV positive subset was included	HI titers pre-vaccination and 28–35 days post-vaccination. Multiple influenza disease endpoints evaluated	Seroprotection to H1N1 93.3%, H3N2 78.0%, and B 96%. Overall vaccine efficacy of preventing confirmed influenza 54.4%. Seroprotection in HIV-infected was 48.6% H3N2 and 68.6% H1N1, but vaccine efficacy was 70.6% in this subset	High levels of seroprotection for HIV-uninfected women post-vaccination. Decreased seroprotection in HIV-infected women but increased vaccine efficacy. Protection from laboratory-confirmed influenza in pregnant women and their infants
Kay et al. (16)	Seasonal IIV, 2012–2013	20 pregnant women, second and third trimesters, 18 non-pregnant women. Significantly fewer pregnant women had received the vaccination in the prior year	HI titers, VMN titers, and plasmablast identification pre- and post-vaccination	Seroprotection H1N1 100%, H3N2 94.4–100%, B 77.8–90.0%. HI and VMN titers were strongly correlated for each strain. Plasmablasts 1.32% pregnant vs. 0.46% control 7 days post-vaccination (*p* = 0.03)	No difference in post-vaccination titers in pregnant vs. control women. Increased fold-changes and decreased pre-vaccine titers in pregnant women. Possibly increased plasmablast induction in pregnant women

Reference	Vaccine, dose	Study participants	Immune assays	Vaccine response	Summary

**IMMUNOGENICITY OF MONOVALENT PH1N1 VACCINE IN PREGNANT WOMEN**					
Zuccotti et al. (24)	MF59 adjuvented pH1N1 monovalent vaccine (7.5 μg)	75 pregnant women (third trimester). Infants were also enrolled through 5 months	HI titers were collected at delivery, 2 months and 5 months post-delivery. No pre-vaccination titer	Seroprotection in 100% of pregnant women at delivery, 2 and 5 months. Seroprotection in 95.6% of infants at delivery and 2 months and 81.2% at 5 months. Infant/maternal antibody ratio of 0.55 at delivery	High rates of seroprotection in pregnant women and their infants following adjuvanted pH1N1 vaccination. Persistent protective antibody levels through 5 months in infants and their mothers
Ohfuji et al. (23)	Two doses of monovalent pH1N1 vaccination (15 μg)	150 pregnant women, all trimesters	HI titers before the first vaccine, 3 weeks after the first vaccine and 4 weeks after the second dose	Seroprotection was observed in 91% after the first dose. No significant change was noted after the second dose	High rates of seroprotection were seen after one dose of the vaccine. Receipt of seasonal influenza vaccination <19 days prior to receipt of the monovalent pandemic vaccination was associated with decreased HI responses
Tsatsaris et al. (15)	Single dose of monovalent pH1N1 vaccine (15 μg)	110 women equally divided between the second and third trimesters. Infant cord bloods collected	HI and VMN titers pre- and post-vaccination	Seroprotection was observed in 98% post-vaccination and 93% of women seroconverted. HI and VMN titers were highly correlated (*r* = 0.96). Maternal and infant cord-blood titers were also correlated (*r* = 0.86)	High rates of seroprotection after a single dose. Women with twins had significantly lower post-vaccination titers. Prior seasonal influenza vaccination was associated with lower fold increase. Significantly higher titers in cord blood suggesting active transport of antibody generated by IIV
Jackson et al. (14)	Two doses of monovalent pH1N1 vaccine at different doses (25, 49 μg)	120 women in the second or third trimester, 60 received the 25 μg and 60 received the 49 μg vaccine. Infant cord bloods collected	HI and VMN pre and post-vaccinations	93% of women met criteria for seroprotection after a single dose. No significant benefit to two doses or the vaccine with increased antigen content. HI and VMN correlation (*r* = 0.81). GMR of cord-blood titer/maternal titer was 1.81 in the 25 μg group and 2.96 in the 49 μg group	High rates of seroprotection after a single dose. No association with vaccine response and prior receipt of seasonal influenza. Significantly higher titers in cord blood suggesting active transport of antibody generated by IIV

*HI, hemagglutinin inhibition assay; VMN, viral microneutralization assay; GMT, geometric mean titer; GMR, geometric mean ratio; Seroprotection, HI titer ≥1:40; Seroconversion, fourfold or greater change in HI titer post-vaccination; IIV, inactivated influenza vaccine*.

Further confirmation of vertical antibody transfer has come from the randomized-controlled trials of influenza vaccine efficacy in pregnant women. In the study performed in Bangladesh, there was no difference in maternal and infant cord-blood HI titers at the time of delivery (22). The infants whose mothers were vaccinated continued to have elevated titers when compared to infants of mothers vaccinated with pneumococcus vaccine through 20–26 weeks of life. The South African study by Madhi et al. also evaluated cord-blood titers compared to maternal titers 1 month after vaccination (30). The ratio of cord blood to maternal titers was 0.7 (0.6–0.8) for pH1N1 and for H3N2 and 0.8 (0.7–0.9) for B/Victoria. Zuccotti et al. also evaluated cord-blood titers following maternal vaccination with an MF-59 adjuvanted pH1N1 monovalent vaccine and found GMT HI titers of 141.8 at birth vs. 257.9 in the mothers, 106.5 at 2 months and 38.3 at 6 months in the infants.

In summary, IIV during pregnancy results in efficient transplacental transfer of the generated antibodies. While, most studies have demonstrated equivalent titers in mothers at delivery and cord-blood samples, some have shown elevated titers in cord blood consistent with active antibody transport (37). While some studies have hinted at lower cord-blood titers among pregnant women vaccinated in the first trimester, it is unclear if this is clinically significant. It has been demonstrated that a 2-week window prior to delivery from the time of influenza vaccination is necessary for placental antibody transfer (38). The maternal antibodies are still present at 3 months post-delivery, and wane slowly thereafter. While maternal antibodies may suppress infant responses to influenza vaccination at 1–2 months of life (39), there is no evidence to date suggesting a decreased response to influenza vaccination at 6 months of life.

## Clinical Efficacy: Maternal

There is clinical data to evaluate the efficacy of influenza vaccination in reducing the incidence of influenza during pregnancy. Two randomized-controlled trials have evaluated both maternal and infant outcomes. In the study by Zaman et al., women vaccinated with IIV during pregnancy had a 35.8% (CI 3.7–57.2) reduction in febrile respiratory illness when compared to pregnant women vaccinated with pneumococcus during pregnancy. In the study by Madhi et al., of 2116 HIV-negative South African pregnant women undergoing seasonal IIV vs. placebo, there was a 50.4% (CI 14.5–71.2) reduction in laboratory-confirmed influenza. This effect was also observed in HIV-infected pregnant women (*p* = 0.03). However, in this trial, there was not a significant difference in the more non-specific diagnosis of influenza-like-illness.

The question of clinical efficacy has also been approached through the use of large health databases. The efficacy of the monovalent pH1N1 vaccine in pregnant women was evaluated through an analysis of Norwegian health registries (40). Approximately 54% of over 113,000 eligible pregnant women received the monovalent pH1N1 vaccination. The risk of receiving a clinical diagnosis of influenza was significantly reduced in this group (adjusted hazard ratio, 0.30; 95% CI 0.25–0.34), suggesting that the monovalent pH1N1 vaccination was efficacious in pregnant women. The efficacy of seasonal influenza vaccination has also been evaluated through analysis of data from a large health plan in the U.S. (41). This was a case–control study over two influenza seasons using a test negative design that estimated a vaccine efficacy of 44% (CI, 5–67%). This was well within the range of efficacy estimates for healthy adults in the same influenza seasons. Thus, both randomized-controlled trials and database analyses suggest that vaccination is efficacious in reducing maternal influenza.

## Clinical Efficacy: Infants

There is even more substantial evidence suggesting that maternal influenza vaccination reduces laboratory-confirmed influenza and influenza related hospitalization in infants of vaccinated mothers. This is critically important because infants <1 year of age, and particularly those <6 months of age, are at very high risk for hospitalization from influenza infection (42). Further, infants <6 months are not vaccinated themselves because IIV does not produce an adequate immune response in this age group, possibly a result of the preexisting maternal antibody (39). Fortunately, two randomized-controlled trials have revealed a significant reduction in clinical and laboratory-confirmed influenza in the infants of vaccinated mothers (21, 30). The study by Zaman et al. of 340 mothers and their infants found that maternal vaccination with IIV was associated with a vaccine efficacy of 63% (95% CI 5–85%) in preventing laboratory-confirmed influenza (21). In addition, they found a significant reduction in respiratory illness with fever, with an associated vaccine efficacy of 29% (95% CI, 7–46). Finally, maternal influenza vaccination was associated with a 42% (95% CI 18.2–58.8) reduction in infant clinic visits. Madhi et al. found that there was a vaccine efficacy rate of 48.8% (95% CI, 11.6–70.4) in preventing RT-PCR-confirmed influenza. However, there was not a difference in infants presenting with influenza-like illness or with any respiratory illness (30).

In addition to the randomized-controlled trials, several other studies suggest a benefit to the infant from maternal influenza immunization. Benowitz et al. performed a matched case–control study with case patients defined as infants under 12 months that were admitted to the hospital due to laboratory-confirmed influenza between October 2000 and April of 2009 (43). For each case, one to two infants who tested negative for influenza were also enrolled. Only 2.2% of the mothers of 91 case subjects aged <6 months had received the influenza vaccination during pregnancy as compared to 19.9% of 156 controls. The adjusted vaccine efficacy was 91.5% (95% CI, 61.7–98.1%; *p* = 0.001). There was not a significant benefit in infants older than 6 months of age, potentially secondary to the waning of maternal antibodies, as infants that were vaccinated were excluded from the study. Poehling et al. performed a similar analysis through use of the New Vaccine Surveillance Network that monitored admission due to influenza among infants in three U.S. counties (44). The study included data from multiple years before the 2009 pandemic and found that infants of vaccinated mothers <6 months of age were 45–48% less likely to be hospitalized for influenza than infants of unvaccinated mothers.

Another group approached this in a prospective fashion evaluating 1169 mother–infant pairs delivering in three consecutive influenza seasons between December 2002 and March 2005 (45). In this study, influenza vaccination did not have an impact of the number of outpatient visits attributable to influenza-like illness. However, it did significantly reduce hospitalizations secondary to influenza-like illness and laboratory-confirmed influenza virus infection by 39 and 41%, respectively. Infants of vaccinated mothers also had significantly higher HI titers to all vaccine strains compared with infants of unvaccinated mothers. Earlier studies by Puck and Reuman had demonstrated that infants with increased cord blood neutralizing antibodies to influenza had delayed infection with influenza, also suggesting a role for maternal antibodies in the prevention of influenza disease in infants (46, 47).

## Conclusion

Overall, the data indicate that pregnant women mount adequate and effective responses to influenza vaccination. There is strong evidence that pregnant women respond at a level that is comparable with other healthy adults. Further, there is also good evidence that protective antibodies are transferred to infants, with the majority of studies indicating that cord-blood titers are equivalent to maternal titers at the time of delivery. Although both second and third trimester vaccinations appear to be equally efficacious, there is less data on first trimester vaccination. Data are mixed relative to whether first trimester vaccination results in diminished cord-blood titers. In some cases, especially following monovalent H1N1 vaccination, cord-blood titers have been consistently higher than maternal titers. The data mirror those of other vaccines that elicit a primarily IgG1 vaccine response, as active placental transfer of antibody through placental Fc receptors primarily occurs with this class of IgG (48). The nature of cellular responses to influenza vaccinations is less well defined; a deficit in interferon production *in vitro* has been observed in pregnant women; however, this effect was rescued by vaccination. Pregnant women have equal and perhaps increased plasmablast induction compared to non-pregnant women following IIV.

The robust immunogenicity of influenza vaccination in pregnancy correlates with clinical efficacy. The vaccine is effective at reducing clinical illness in pregnant women at a level on par with that observed for non-pregnant healthy subjects. In addition, there is a clear benefit to infants of vaccinated mothers up through 6 months of age, presumably through active transport of maternal antibody. While not addressed in this review, there is substantial evidence that influenza vaccination is safe for pregnant women and the fetus with no evidence that immunization increases the risk of preterm delivery or other adverse pregnancy outcomes (19, 49–52). In summary, influenza vaccination is both highly immunogenic and clinically beneficial for pregnant women and their infants.

## Conflict of Interest Statement

The authors declare that the research was conducted in the absence of any commercial or financial relationships that could be construed as a potential conflict of interest.
